# Supplementary material for: A set of multi-entry identification keys to African frugivorous flies (Diptera, Tephritidae)
Source: Zookeys. 2014 Jul 24;(428):97–108. doi: 10.3897/zookeys.428.7366 (PMC4143993; doi:10.3897/zookeys.428.7366)
Supplement: Supplementary material 4 — Key to Capparimyia [file zookeys-428-097-s004.zip › SF4_ZooKeys_key to Capparimyia/lucid_player/help/help/finding_features.htm]

Finding features, states and entities


# Finding features, states and entities

To search for a feature or state, activate one of the *Features*
panels by clicking within it, then choose *Find feature* or *state*.
To search for an entity, activate one of the *Entities* panels by
clicking within it, then choose *Find entity.*

In the *Find* dialog, type the text to search for in the *Find
What* box. Use the *Up* or *Down* buttons to specify a direction
to search from the currently selected item. If Match Case is checked,
the search will be case-sensitive (that is, an item called *Item*
will not be found if the search text is item). If *Match* whole word
only is checked, the search will match only whole items (that is, an item
called *Item One* will not be found if the search text is *Item*
or *One)*.

The *Find* function can also be invoked using the keyboard shortcut
*Ctrl+F* or the *Find*...  button on the main
button bar.
